# Overcoming TRAIL Resistance for Glioblastoma Treatment

**DOI:** 10.3390/biom11040572

**Published:** 2021-04-14

**Authors:** Longfei Deng, Xuan Zhai, Ping Liang, Hongjuan Cui

**Affiliations:** 1 Cancer Center, Medical Research Institute, Southwest University, Chongqing 400716, China; lfdeng@swu.edu.cn; 2 Department of Neurosurgery, Children’s Hospital of Chongqing Medical University, Chongqing 400014, China; zhaixuan@163.com; 3 State Key Laboratory of Silkworm Genome Biology, Southwest University, Chongqing 400716, China

**Keywords:** TRAIL, death receptor, resistance, glioblastoma, apoptosis

## Abstract

The tumor necrosis factor (TNF)-related apoptosis-inducing ligand (TRAIL) shows a promising therapeutic potential in cancer treatment as it exclusively causes apoptosis in a broad spectrum of cancer cells through triggering the extrinsic apoptosis pathway via binding to cognate death receptors, with negligible toxicity in normal cells. However, most cancers, including glioblastoma multiforme (GBM), display TRAIL resistance, hindering its application in clinical practice. Recent studies have unraveled novel mechanisms in regulating TRAIL-induced apoptosis in GBM and sought effective combinatorial modalities to sensitize GBM to TRAIL treatment, establishing pre-clinical foundations and the reasonable expectation that the TRAIL/TRAIL death receptor axis could be harnessed to treat GBM. In this review, we will revisit the status quo of the mechanisms of TRAIL resistance and emerging strategies for sensitizing GBM to TRAIL-induced apoptosis and also discuss opportunities of TRAIL-based combinatorial therapies in future clinical use for GBM treatment.

## 1. Introduction

Programed cell death via apoptosis is an integral part of normal physiological mechanisms to eradicate unwanted or damaged cells and also functions as a natural barrier against cancer development and progression [1]. However, tumor cells resist cell death through evolving multiple strategies to attenuate or circumvent apoptosis, including short-circuiting the extrinsic apoptosis pathway which is activated by cell death receptors upon binding to cognate ligands [2], such as the Fas ligand (FasL/Apo1L/CD95L), Apo 3 ligand (Apo3L), TNF-alpha (TNFα), and TNF-related apoptosis-inducing ligand (TRAIL, also designated as TNFSF10 and APO2L) [3]. Among them, TRAIL appears to be the most promising candidate therapeutic for targeted therapy for cancer in clinic since it preferentially initiates apoptosis in a broad variety of tumor cells without overt cytotoxicity to normal cells both in vitro and in vivo [4,5]. TRAIL is a pro-apoptotic cytokine acting as an extracellular signal to trigger apoptosis via binding to death receptors TRAIL-R1 (TRAIL receptor 1) [6] and TRAIL-R2 [7,8,9], which transmit death signals through a cytoplasmic “death domain” motif, thereby inducing the formation of the pro-apoptotic death-inducing signaling complex (DISC) and the downstream activation of apoptotic cascade to execute cell apoptosis [10]. However, the clinical response to TRAIL and TRAIL-based therapeutics is unsatisfactory in most cancers, with glioblastoma multiforme (GBM) being particularly resistant [11,12], defining an imperious need to thoroughly resolving the mechanisms of TRAIL resistance. Recent studies have revealed novel regulators of TRAIL-induced apoptosis in GBM and focused on seeking more efficient TRAIL delivery systems and combination therapies with appropriate sensitizing drugs. This review attempts to summarize the updated understanding of TRAIL resistance mechanisms in GBM and strategies to overcome them and also discusses the promising future prospects of translating TRAIL into clinical use for treating GBM.

## 2. Targeting the TRAIL Receptor/Ligand System: An Emerging Opportunity for GBM Treatment

### 2.1. GBM Treatment Is a Formidable Challenge in Clinical Oncology

GBM is a highly malignant primary brain tumor and the most aggressive type of gliomas arising from the central nervous system (CNS) [13]. Despite being one of the most studied tumors, the therapeutic modalities for GBM have changed little in the past decade, mainly including neurosurgical debulking aiming for maximal tumor resection, followed by concurrent fractionated local radiation and adjuvant temozolomide chemotherapy to combat the residual tumors [14,15,16]. However, as a predominant adult brain tumor and one of the most lethal human malignancies, GBM is an incurable disease with a poor prognosis and nearly universal relapse. Patients diagnosed with GBM have a median overall survival (mOS) of 16–21 months, with only 43% of them surviving for 2 years [17,18,19].

Apart from tumor-treating fields, no other therapeutic interventions used in the new diagnosis or at recurrence exhibit possibilities to prolong overall survival in randomized trials [20]. Several disadvantages impede the development of novel therapies for GBM, such as GBM’s infiltrative nature, the blood–brain barrier, tumor heterogeneity, unique tumor microenvironments, and the high metastatic and angiogenic potential of the tumors [14]. Currently, the effective treatment options for treating GBM are still lacking, rendering the treatment of GBM as one of the most difficult challenges in clinic oncology [21]. Although thousands of GBM-related studies are published each year, more translational studies on developing innovative therapeutic strategies are urgently needed for improving the poor outcome of GBM treatment.

### 2.2. TRAIL-Induced Apoptotic Signaling

Tumor cells including GBM have evolved multiple strategies to escape programmed cell death, underscoring that the reactivation of cell death programs, which are frequently inactivated in GBM, is a reasonable avenue to overcome this Achilles′ heel of GBM [22]. Among the forms of programmed cell death, the most extensively studied is apoptosis. Two known major signaling pathways, i.e., the death receptor (extrinsic) pathway and the mitochondrial (intrinsic) pathway, have been delineated to regulate the apoptotic process [23]. The activation of the mitochondrial (intrinsic) pathway requires a mitochondrial-to-cytosol release of proteins such as cytochrome c or Smac to initiate apoptosis through engaging caspase activation. Although the eventual caspase activation is a shared apoptotic mechanism, the death receptor (extrinsic) pathway differs in part by launching apoptosis via cell surface death receptors bound to cognate death receptor ligands [24]. Death receptors belong to the tumor necrosis factor (TNF) receptor superfamily, such as TNF-receptor 1 (TNFR1), Fas/APO-1/CD95, death receptor 4/TRAIL-R1, and death receptor 5/TRAIL-R2, which are ligated by the corresponding death receptor ligands, namely, TNFα, FasL, and TRAIL, when the death receptor-mediated apoptosis is activated [25].

TRAIL was identified independently by two groups in 1995 and 1996 as a 281-amino acid type II transmembrane protein in the human form or 291-amino acid protein in the murine form and found to rapidly induce extensive apoptosis in a variety of transformed cell lines [26,27]. One year later, two TRAIL receptors, TRAIL-R1 and TRAIL-R2, were defined to mediate the pro-apoptotic effect of TRAIL on tumor cells [28]. In 1999, the tumoricidal activity and safety of recombinant TRAIL on normal cells was demonstrated in vivo [4,5]. These serial groundbreaking discoveries have aroused great interest in ascertaining the molecular mechanisms of TRAIL/TRAIL-R1/R2 axis-induced apoptosis and developing TRAIL-R agonists for cancer therapy.

The signal transduction of TRAIL-induced apoptosis is becoming increasingly clear with extensive investigations. It is now known that upon stimulation by TRAIL, trimeric TRAIL-crosslinked TRAIL-R1/R2 undergo homotrimerization and employ their intracellular death domains (DDs) to recruit the FAS-associated death domain protein (FADD), which subsequently recruits pro-caspase-8 through the death effector domains (DEDs), collectively forming the death-inducing signaling complex (DISC) to activate the pro-caspase-8. The activated dimeric caspase-8 is released to cleave and activate the effector caspase-3 that is sufficient to trigger extrinsic apoptosis in some cells (type I). Whereas in other cells (type II), the activation of the mitochondrial pathway is also required for apoptosis induction. Under this circumstance, caspase-8 cleaves the Bid (BH3 interacting-domain death agonist), and then BAK (BCL2 antagonist/killer) and BAX (BCL2 associated X) oligomerize in the outer mitochondrial membrane to form lipid-containing pores. Consequently, cytochrome c is released, together with Apaf-1 (apoptotic peptidase activating factor 1) and pro-caspase-9, assembling an apoptosome, which in turn enhances caspase-3 cleavage and the activation of other effector caspases, resulting in cleavage of a plethora of cellular proteins and ultimately the execution of apoptosis [29,30,31]. Hence, both branches of the apoptosis pathway can be activated by TRAIL, dependent on caspase-8-mediated cleavage, highlighting a central role of caspase-8 in transducing TRAIL-induced apoptotic signaling. This crosstalk of apoptotic signaling pathways generates a complex network of TRAIL-induced apoptosis programs. Whereas, unlike the pro-apoptotic receptors TRAIL-R1/R2, both TRAIL-R3 and TRAIL-R4 act as “decoys” to negatively regulate TRAIL-induced apoptosis by competing with TRAIL-R1/R2 [32,33], further complicating the regulation of TRAIL-induced apoptosis.

In addition to apoptotic signaling, it is well characterized that TRAIL can trigger various non-apoptotic signaling pathways to induce a plethora of biological responses. A typical example is that TRAIL activates the receptor-interacting serine/threonine protein kinase 1 (RIPK1) and RIPK3 signaling to induce necroptosis [34], another form of cell death. Other pathways that can be activated by TRAIL, such as the nuclear factor-κB (NF-κB), JUN N-terminal kinase (JNK), p38 MAPK pathways and oncogenic KRAS signaling, and the physiological functions have been thoroughly reviewed elsewhere [30]. The intricate link between these TRAIL-induced molecular events and apoptosis programs is not fully clear and warrants further interrogations, which would help the development of TRAIL-based cancer therapies.

### 2.3. TRAIL Receptor/Ligand System Is a Promising Therapeutic Target for GBM

Among the death receptor-ligand systems, the TNFR1-TNFα and CD95-FasL axes are excluded from further therapeutic exploitations, owing largely to the lethal toxicity of TNF or CD95 agonists [30]. On the other hand, however, pre-clinical studies have shown that TRAIL administration has preferential tumoricidal activity on GBM without detectable toxicity to normal brain tissue [11,35]. Bortezomib is a proteasome inhibitor proven well tolerated and safe in combination with temozolomide and radiation in GBM treatment in early clinical trials [36,37]. Remarkably, TRAIL was found to cooperate with bortezomib to augment apoptosis in cultured GBM cells and patient-derived GBM stem cells and retard tumor growth in vivo [38]. This potential clinical relevance is reinforced by studies demonstrating that the combined administration of TRAIL and systemic temozolomide has a synergistic antitumor effect against GBM cells in vitro [39] and also prolongs survival in an intracranial GBM xenograft model [40]. Importantly, ONC201, a TRAIL-inducing compound, has proven well tolerated, capable of passing the blood–brain barrier, and has shown preliminary signs of efficacy in 17 patients with aggressive and recurrent GBM in a phase 2 clinical trial [41]. These results are very encouraging and tests in an expanded cohort of GBM patients are ongoing [42,43]. Given this promising therapeutic impact of the TRAIL receptor/ligand system for GBM treatment, more clinical trials are expected to launch in the future to test TRAIL-based combinatory modalities for treating GBM.

For other solid tumors, however, dissatisfactory results from clinical trials of recombinant TRAIL and agonist antibodies against TRAIL-R1/2 have been obtained, and the drug’s short half-life, poor pharmacokinetics, and resistance are thought to be the major contributors [44]. Regardless of the failure of past endeavors, the recent development of novel methods, such as protein modification, combinatorial therapy, and TRAIL-based gene delivery, paves a new avenue to improve the efficacy of TRAIL-based therapy for generating robust anticancer activities [45]. Currently, combinatory therapies consisting of novel recombinant TRAIL or agonist antibodies against TRAIL-R1/2 are being actively tested in several clinical trials, which are summarized in a recent review [44]. These progresses will provide valuable lessons for future clinical trials in GBM therapy.

### 2.4. The Expression Patterns of TRAIL/TRAIL Death Receptors and Implications in GBM Treatment

Studies using different analyses have shown that TRAIL receptors, including TRAIL-R1 and TRAIL-R2, as well as “decoy” receptors TRAIL-R3 and TRAIL-R4 that lack the cytoplasmic “death domain” to induce the apoptotic pathway, are all expressed with varying degrees in several GBM cell lines [39,46,47]. A constant transcriptional co-expression of TRAIL, TRAIL-R1, TRAIL-R2, and TRAIL-R3 was also confirmed in human primary GBM [48]. In addition, slightly diffused cytoplasmic and a stronger membranous staining of TRAIL-R1, TRAIL-R2, and TRAIL were visualized on GBM tumor specimens, and the expression of TRAIL-R1 and TRAIL-R2 was reported as an independent prognostic factor for the survival of patients with primary GBM, implicating both of them as possible targets for TRAIL therapy [49]. It is also proposed that TRAIL-R2 appears more important as a druggable target due to its higher level than TRAIL-R1 [49]. Noteworthily, both a homotrimeric type II transmembrane TRAIL (memTRAIL) and a soluble form of TRAIL (sTRAIL) efficiently activate TRAIL-R1 to induce apoptosis even at low concentrations, but only very high levels of them are able to activate TRAIL-R2 [50,51,52]; therefore, the relatively lower expression of TRAIL-R1 might help GBM tumors to evade apoptosis upon TRAIL/TRAIL-R1 binding. From this perspective, TRAIL-R1 may also be a significant target for TRAIL-based therapy of GBM.

Yet, some in vitro observations in GBM cell lines have shown that the levels of TRAIL receptors are not necessarily correlated with TRAIL sensitivity [53,54], suggesting TRAIL receptor expression alone is not adequate to determine TRAIL sensitivity, but rather, alterations in the pathway that links TRAIL receptor activation to the apoptotic machinery also play an indispensable role. Nevertheless, it is tempting to assess whether a correlation exists between TRAIL receptor expression and TRAIL sensitivity in GBM tumors in clinical scenarios. It is also established that FasL expressed on the surface of GBM tumors binds to Fas and leads to apoptosis of invading immune cells, therefore enabling GBM cells to maintain immune privilege and elude immune attacks [55]. It is plausible that analogous to FasL, the membranous expression of TRAIL in GBM tumors represents a possible tumor defense mechanism against killing by immune cells. Therefore, circumventing TRAIL-mediated immunosuppressive hurdles is presumably another strategy to optimize TRAIL-based therapeutics for GBM patients.

## 3. Aberrations in TRAIL-Induced Apoptotic Signaling in GBM

Cancer cells, including GBM, have evolved multiple strategies to rewire TRAIL-induced apoptotic signaling in order to evade apoptosis. For example, GBM cells display very low level of caspase-8, which is correlated with resistance to TRAIL-induced apoptosis. Additionally, in tumors obtained from GBM patients, caspase-8 expression is also very low, suggesting that the TRAIL pathway may not be functional in GBM due to insufficient caspase-8 activation [56]. Moreover, the cellular FLICE ((Fas-associated death domain–like IL-1β–converting enzyme)) inhibitory protein (c-FLIP) competes with caspase-8 for binding to FADD and therefore suppresses DISC activation, and in human GBM, c-FLIP expression is upregulated to resist TRAIL-induced apoptosis [57]. In addition, the anti-apoptotic proteins, such as Bcl-2 and Bcl-xL, are frequently overexpressed in GBM [58,59]. Studies have demonstrated that Bcl-2 ectopic overexpression inhibits TRAIL-induced apoptosis in GBM cell lines [60]. In contrast, specific Bcl-2 inhibitor and Bcl-2/Bcl-xL inhibitor potently reactivate TRAIL-induced apoptosis in GBM cells [54]. Moreover, Apaf-1 participates in the assembly of a functional apoptosome, but Apaf-1 is inactivated by high frequency of loss of heterozygosity at chromosome 12q22–23 in GBM [61]. In human GBM cell lines, multiple simultaneous genomic alterations in TRAIL-R1, TRAIL-R2, caspase-8, Bid, and Smac loci were found to contribute to TRAIL resistance [62]. These aberrations and others (summarized in Table 1) in TRAIL-induced apoptotic signaling should be taken into consideration and can be regarded as targets in the development of therapeutic approaches to reactivate apoptosis signaling networks for eradicating GBM.

## 4. Advances in Mechanisms of TRAIL-Induced Apoptotic Signaling in GBM

**Figure 1:**
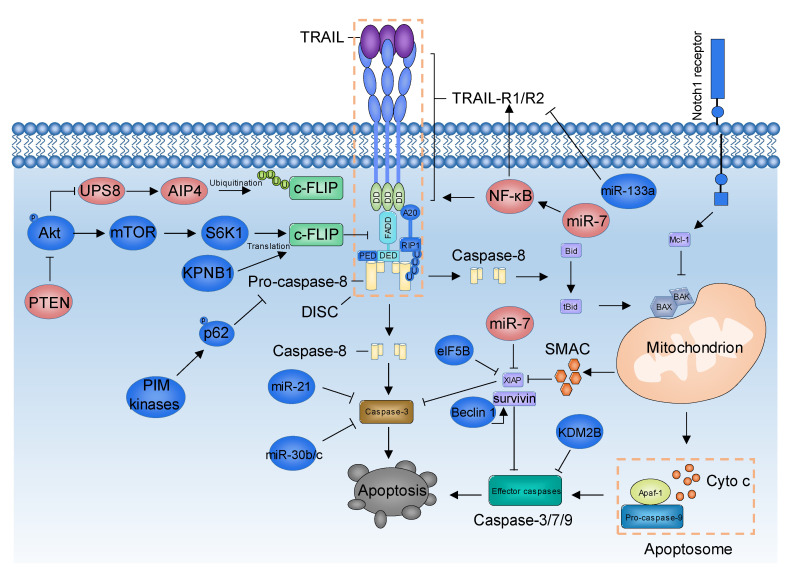
Updated mechanisms that regulate TRAIL-induced apoptotic signaling in glioblastoma multiforme (GBM). Proteins depicted in deep blue are negative regulators of TRAIL-induced apoptosis, whereas proteins depicted in pink are positive regulators of TRAIL-induced apoptosis in GBM. TRAIL, tumor necrosis factor-related apoptosis-inducing ligand; TRAIL-R1/R2, TRAIL receptor1/recptor2; DD, death domain; FADD, FAS-associated death domain protein; DED, death effector domain; PED, phosphoprotein enriched in diabetes; c-FLIP, cellular FLICE inhibitory protein; DISC, death-inducing signaling complex; RIP1, receptor interacting protein kinase 1; A20, E3 ubiquitin ligase A20/TNFAIP3; AIP4, atrophin-interacting protein 4; PIM, proviral integration site in Moloney murine leukemia virus; NF-κB, nuclear factor-κB; SMAC, second mitochondria-derived activator of caspase; Apaf-1, apoptotic peptidase activating factor 1; KDM2B, lysine-specific demethylase 2B; Cyto c, cytochrome c.

In addition to well-established TRAIL-induced apoptotic signaling, increasing evidence has unraveled novel mechanisms that either promote or inhibit TRAIL-induced apoptosis in GBM (Figure 1), offering potential therapeutic targets for overcoming TRAIL resistance.

### 4.1. Mechanisms That Promote TRAIL-Induced Apoptosis in GBM

#### 4.1.1. NF-κB

Since resisting apoptosis is a hallmark of human cancers, it may not be so surprising that few mechanisms are dedicated to promoting TRAIL-induced apoptosis in GBM. One study has reported that nuclear factor-κB (NF-κB) activation via overexpression of constitutively active IκB kinase complex (IKK) β (IKK-EE) significantly increases TRAIL-mediated apoptosis. Conversely, the inhibition of NF-κB by overexpression of the dominant-negative IκBα superrepressor (IκBα-SR) decreases TRAIL-induced apoptosis in GBM cells [74]. Mechanistically, the inhibition of NF-κB reduces the recruitment of FADD and caspase-8 and formation of DISC upon stimulation of TRAIL receptors, which restrains the TRAIL-mediated activation of caspases, loss of mitochondrial potential, and cytochrome c release, resulting in decreased TRAIL-induced apoptosis in GBM cells [74]. These findings reveal a pro-apoptotic role of NF-κB in TRAIL-induced apoptosis in GBM cells by facilitating DISC formation. Similarly, NF-κB has also been noticed to exert a pro-apoptotic role in DNA damage-triggered apoptosis in GBM cells [75]. Paradoxically, however, NF-κB inhibition enhances TRAIL-induced apoptosis in mouse embryonic fibroblasts and neuroblastoma cells [74,76] and is also implicated in lovastatin-sensitized TRAIL-induced apoptosis in resistant GBM cells via upregulation of TRAIL-R2 level [77]. Nevertheless, an early study showed that specific inhibition of NF-κB by overexpression of IκBα-SR had no significant impact on GBM cell apoptosis induced by TRAIL, implying that the characteristic anti-apoptotic function of NF-κB in many cancers is not a primary feature for GBM [78]. These cell type-dependent findings in GBM cells have implications for designing strategies of manipulating NF-κB activity to overcome TRAIL-induced apoptosis resistance in GBM.

#### 4.1.2. miR-7

The X-linked inhibitor of apoptosis (XIAP) exerts anti-apoptotic functions via inhibiting the activation of caspases. As expected, inhibition of XIAP with Embelin enhances TRAIL-mediated apoptosis in GBM cells [79]. A genome-wide analysis has identified that miR-7 critically promotes TRAIL-induced apoptosis in GBM cells through targeting XIAP, and combining miR-7 overexpression with TRAIL leads to a synergistic tumor suppression effect both in vitro and in vivo [80]. Consistent with this study, another investigation has demonstrated that miR-7 expression in GBM cells results in an upregulation of TRAIL-R2 via activating NF-κB, ultimately priming resistant GBM cells to TRAIL-induced apoptosis. Further, miR-7 overexpression significantly decreases tumor growth and potentiates TRAIL activity to eradicate GBM xenografts formed by patient-derived primary GBM stem cell (GSC) lines and improves mouse survival [81]. These observations together identify miR-7 as a novel positive regulator of TRAIL-induced apoptosis and provide miR-7 as a promising therapeutic candidate for reducing TRAIL resistance in GBM.

### 4.2. Mechanisms That Inhibit TRAIL-Induced Apoptosis in GBM

#### 4.2.1. c-FLIP

Similar to most tumors, GBM tumors display a range of TRAIL sensitivity, but the majority harbor innate resistance to TRAIL-induced apoptosis, which prevents the clinical application of TRAIL [82]. The mechanisms underlying GBM resistance to TRAIL are still not completely understood, but many progresses in this field have been witnessed over the past two decades. One study has shown that the Akt-mTOR-S6K1 pathway enhances translation of c-FLIP that blocks caspase-8 activation, thereby conferring TRAIL resistance to GBM cells. Reversely, inhibition of mTOR or S6K1 decreases c-FLIP protein level and suppresses TRAIL resistance. Moreover, in xenografted human GBM, the activation status of the PTEN-Akt-mTOR pathway distinguishes the inherent TRAIL-sensitive tumors from those sensitized by rapamycin, an mTOR inhibitor [57]. Although further studies are needed to examine the correlation between mTOR pathway status and clinical response to TRAIL, this study suggests that the mTOR pathway is an important mediator of TRAIL resistance in GBM. This also provides a rationale for a combinatorial therapy of TRAIL with mTOR inhibitors in GBM treatment, such as CCI-779, which is widely used clinically and possesses activity against PTEN-deficient GBM tumors [83]. Further studies have demonstrated that the PTEN-Akt pathway also controls c-FLIP ubiquitination via the ubiquitin-specific protease 8 (USP8) and an E3 ubiquitin ligase atrophin-interacting protein 4 (AIP4), leading to prolonged c-FLIP half-life and increased TRAIL resistance in GBM cells [84,85]. The ubiquitin control pathway described in these works broadens the regulatory mechanisms of TRAIL resistance.

A recent study reveals that genetic or pharmacological inhibition of karyopherin β1 (KPNB1) potentiates TRAIL-induced apoptosis selectively in GBM cells partially through accelerating caspase-8 cleavage via downregulating c-FLIP [86], proposing that the combination of KPNB1 inhibitor and TRAIL could rewire the TRAIL receptor signaling and abrogate TRAIL resistance. Since KPNB1 inhibitor ivermectin has been proven safe at a high-dose [87], its combination with TRAIL may be a promising candidate for anti-GBM clinical trials. Together, these researches uncover diverse mechanisms of upregulating levels of c-FLIP for conferring TRAIL resistance to GBM cells. Accordingly, techniques such as targeted c-FLIP degradation via proteolysis-targeting chimera (PROTAC) may represent a potential strategy to override TRAIL resistance in GBM [88]. 

#### 4.2.2. Caspase-8 Inhibitors

As described, caspase-8 is a master regulator in TRAIL-induced apoptosis. Understandably, genomic alterations in CASP8 contribute to TRAIL resistance in GBM cells [62]. In GBM-derived CSCs, the loss of the CASP8 locus in the 2q33–34 region causes the lack of caspase-8 expression and TRAIL resistance [89]. These findings not only shed light on genomic mechanisms in GBM resistance to TRAIL-induced apoptosis, but also advise future clinical trials to consider genomic analysis of GBM tumors for identifying CASP8 gene status and utilize it as a genomic marker to predict the responsiveness of GBM to TRAIL therapies.

Except canonical regulators of caspase-8 activation during TRAIL-induced apoptotic signaling, recent studies have unveiled some previously unprecedented regulators of caspase-8 activation in GBM and inspired new insights for designing TRAIL-based therapy. For example, it has been proven that the knockdown of PIM kinases decreases phosphorylation of p62 and sensitizes TRAIL-induced apoptosis via enhanced caspase-8 recruitment to and activation at the DISC; in line with this, p62 ablation facilitates TRAIL-induced caspase-8 activation, revealing an inhibitory role of p62 in TRAIL-mediated apoptosis in GBM [90]. Thus, PIM kinases mediate resistance of GBM cells to TRAIL by a p62-dependent mechanism, suggesting that targeting PIM kinases in combination with TRAIL may represent new therapeutic strategies against GBM. Although the first PIM inhibitor SGI-1776 is withdrawn from clinical trials due to cardiac toxicity [91], it has helped in accelerating the discovery of novel PIM inhibitors in recent years, and several other candidates are currently tested in clinical trials for the treatment of cancers, including GBM [92]. 

Another example is A20 ubiquitin ligase, which was illustrated to mediate ubiquitination of RIP1, through which it inhibits caspase-8 dimerization and cleavage and TRAIL-induced apoptosis in tumor-initiating cells isolated from GBM patients [93]. A20 is highly expressed in GBM and forms an assembly complex together with TRAIL-R2 and RIP1; thus, A20 may serve as another potential therapeutic target to overcome TRAIL resistance in GBM through enhancing caspase-8 activation.

#### 4.2.3. DISC Modification

DISC formation is a key upstream event in TRAIL-induced apoptotic signaling. A recent insight into this mechanism is provided by a study manifesting that in TRAIL-sensitive GBM cells, TRAIL-R2 is the only consistently expressed functional receptor that, upon TRAIL binding, homotrimerizes and recruits FADD and caspase-8 for assembling the DISC in the lipid rafts of plasma membrane, wherein caspase-8 is cleaved and initiates apoptosis. However, in non-raft fractions of plasma membrane of TRAIL-resistant GBM cells, TRAIL-R2-mediated DISC is modified by RIP, c-FLIP, and PED/PEA-15, resulting in caspase-8 cleavage inhibition. On the contrary, silencing RIP, c-FLIP, or PED/PEA-15 redistributes the DISC from non-rafts to lipid rafts, thereby eliminating caspase-8 cleavage inhibition and TRAIL resistance [94]. Given this line of evidence, targeting these intracellular adaptors from the upstream event of DISC modification could therefore represent a novel tactic to eliminate TRAIL resistance in human GBM.

#### 4.2.4. miRNAs

miRNAs also attract attention as candidates to lower TRAIL resistance. miR-21 is elevated in GBM and its knockdown increases apoptotic activity. It has been found that the combined suppression of miR-21 with a secretable TRAIL causes an increase in synergistic apoptosis in human GBM cells in vitro and in vivo [95]. In spite of unknown effector targets, this study implicates miR-21 as a target for TRAIL-based therapies in GBM. Additionally, high expression levels of miR-21 and miR-30b/c were shown to be required to maintain a TRAIL-resistant phenotype in GBM cells. These miRs perform this role partially by modulating caspase-3 expression and the TRAIL-induced apoptotic program, making them promising therapeutic targets for antagonizing TRAIL resistance in GBM [96]. Another study has discovered that miR-133a dramatically promotes TRAIL resistance in vitro and in vivo by suppressing TRAIL-R2 expression, implying that silencing of this miRNA may sensitize GBM cells to TRAIL-induced apoptosis [97]. In view of these encouraging findings showing that selective miRNA antagonism sensitizes GBM tumors to TRAIL administration, more potential miRNAs involved in regulation of TRAIL resistance should be identified in future studies to increase the therapeutic response of TRAIL.

#### 4.2.5. Others

The alteration of tumor suppressor p53 is the most common molecular abnormality in GBM, which has been demonstrated in 60–70% cases of GBM patients [98]. The relationship between TRAIL sensitivity and p53 status in GBM has been partly clarified by a study using endogenous and inducible wild-type p53 GBM cell lines, which argues for a protective role of p53 against TRAIL-induced apoptosis [99]. This exploration suggests that p53 functions to confer TRAIL resistance to GBM, implying that TRAIL administration may possess more potent antitumoral activity toward p53-deficient GBM tumors.

An interaction was reported between Beclin 1, a key regulator of autophagy, and survivin that belongs to a member of the anti-apoptotic protein family. Further, Beclin 1 knockdown sensitized GBM cells to TRAIL-induced apoptosis, which was antagonized in the presence of survivin introduction, suggesting that Beclin 1 enhances TRAIL resistance in GBM cells through maintaining the level of survivin [100]. These results point to a possible mechanism of a crosstalk between autophagy and TRAIL-induced apoptosis. It has been demonstrated that TRAIL can induce cytoprotective autophagy, and blocking autophagy via silencing Beclin 1 effectively increases TRAIL-induced apoptotic cytotoxicity in different human cancer cells [101]. Supposedly, Beclin 1-mediated autophagy serves as another positive regulator for developing TRAIL resistance in GBM. Upregulating the anti-apoptotic proteins to help GBM cells survive the TRAIL insults is not limited to the action of Beclin 1. One study has shown that the Notch1 receptor promotes the survival of GBM cells by upregulating the anti-apoptotic Mcl-1 protein, and conversely, the inhibition of Notch1 pathway sensitizes GBM cells to TRAIL-induced apoptosis. Therefore, targeting Notch1 might represent a promising novel strategy in GBM treatment [102].

Most chemotherapies are designed to destroy cancer cells by inducing DNA damage, which may be repaired by the intrinsic DNA damage response machinery, and as a result, cancer cells will survive [103]. Remarkable differences have been observed in levels of proteins pivotal for DNA damage response between TRAIL-sensitive and -resistant GBM cells, such as ATM and CHK2. Therapies that inhibit CHK2 levels in GBM may enhance the efficacy of TRAIL treatment, hinting that DNA damage signaling pathways might contribute to TRAIL resistance and that targeting DNA repair factors is a strategy to overcome TRAIL resistance of GBM [104].

TRAIL pro-apoptotic signaling is subjected to epigenetic regulation [105]. In GBM cells, one study has described that silencing of KDM2B, an H3K36-specific demethylase, significantly enhances TRAIL-induced apoptosis under in vitro and in vivo settings. The underlying molecular mechanisms are multidimensional, which include the derepression of pro-apoptotic genes Harakiri, caspase-7, and TRAIL-R1 and the repression of anti-apoptotic genes. These findings identify KDM2B as a novel regulator in TRAIL resistance in GBM and show that the key TRAIL-induced apoptotic components are under epigenetic control of KDM2B [106]. Another study has reported that the loss of caspase-8 due to methylation of promoter reaches more than 50% within 76 patients with GBM. Moreover, similar to CASP8, more than 40% of GBM cell lines display significant methylation in TRAIL-R1 gene promoter [63], possibly revealing an extensive epigenetic regulation in TRAIL resistance in GBM. These observations may inspire further investigations to elucidate how TRAIL resistance is regulated by epigenetic regulation and exploit the findings to improve the effectiveness of TRAIL therapy.

During cellular stress, the eukaryotic initiation factor 5B (eIF5B) promotes the translation of mRNA encoding the anti-apoptotic factor XIAP [107]. A recent study has shown that the depletion of eIF5B sensitizes GBM cells to TRAIL-induced apoptosis by inhibiting the translation of several mRNAs encoding the anti-apoptotic proteins XIAP, Bcl-xL, cIAP1, and c-FLIPS, indicating that eIF5B allows GBM cells to evade TRAIL-induced apoptosis by promoting the translation of pro-survival proteins [108]. Therefore, eIF5B represents a novel target to sensitize GBM cells to pro-apoptotic TRAIL treatment.

## 5. Sensitizing GBM to TRAIL-Induced Apoptosis

**Figure 2:**
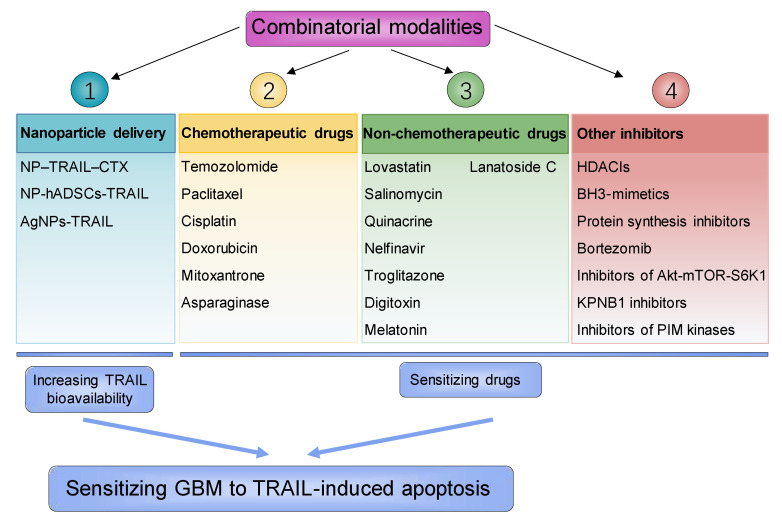
Strategies for sensitizing GBM to TRAIL-induced apoptosis. NP–TRAIL-CTX, nanoparticle coated with chitosan-polyethylene glycol-polyethyleneimine copolymer and chlorotoxin; NP-hADSCs-TRAIL, nanoparticle-engineered human adipose-derived stem cells overexpressing TRAIL; AgNPs-TRAIL, silver nanoparticles-TRAIL; HDACIs, histone deacetylase inhibitor; BH3-mimetics, Bcl-2 homology 3 mimetics; KPNB1, karyopherin β1.

To maximize the potential of TRAIL in treating GBM, most research has focused on developing methods to sensitize GBM to TRAIL treatment through two major directions: increasing TRAIL bioavailability via constructing efficient TRAIL delivery system and enhancing TRAIL tumoricidal activity through combining sensitizing drugs (Figure 2).

### 5.1. Nanoparticle Delivery

The clinical application of TRAIL is largely hindered by its short serum half-life and lack of efficient delivery approaches. In recent years, developing nanoparticles as carriers in gene therapy has been considered as an effective approach to increase TRAIL delivery to tumors as transfected cells will specifically secrete TRAIL into the tumor microenvironment. However, another huge obstacle for gene delivery to GBM in the brain is to cross the blood–brain barrier, and most delivery vehicles fail to generate high gene transfection efficiency in vivo [109]. By developing a targeted iron oxide nanoparticle coated with chitosan-polyethylene glycol-polyethyleneimine copolymer and chlorotoxin, one study has found that this delivery system successfully delivers TRAIL into human GBM cells and induces secretion of TRAIL in vitro and in vivo, resulting in near-zero tumor growth and induces apoptosis in tumor tissue [110]. This study suggests that nanoparticle-mediated TRAIL delivery can serve as a potential targeted therapeutic for more efficient TRAIL delivery to GBM. A similar concept has been applied to human adipose-derived stem cells (hADSCs), in which polymeric nanoparticles, as a drug-delivery vehicle, mediate the overexpression of TRAIL for targeting and eradicating GBM cells in vivo and prolong animal survival [111]. A recent study also reveals that TRAIL sensitivity in GBM cells can be enhanced by conjugation of TRAIL with silver nanoparticles, further supporting nanoparticle delivery to be a promising therapeutic approach to bypass consumption of TRAIL in circulation and effectively increase the TRAIL dose in tumor lesions for sensitizing TRAIL resistance [112].

### 5.2. Combination with Chemotherapeutic Drugs

Most chemotherapeutic drugs kill cancer cells predominantly by triggering the apoptotic program. Increasing evidence has shown that several chemotherapeutic drugs treated in combination with TRAIL can result in the reversal of GBM resistance to TRAIL-mediated apoptosis. For example, combined TRAIL plus paclitaxel have cooperative anti-GBM efficacy in vivo, particularly with no discernable toxicity to normal tissue [113]. Analogically, co-delivery of the TRAIL gene also enhances the antitumor activity of paclitaxel against GBM cells in vitro and in vivo [114]. Except paclitaxel, a synergistic anti-GBM effect has been validated between TRAIL and cisplatin, as evidenced by cisplatin-enhanced sensitivity of GBM cells to adenovirus-delivered TRAIL [115], and cisplatin-restored activation of the TRAIL apoptotic pathway in GBM-derived stem cells [116]. Moreover, doxorubicin and mitoxantrone were also identified as TRAIL-sensitizing agents for GBM [117,118]. Interestingly, L-asparaginase, a metabolic enzyme used in the treatment of acute lymphatic leukaemia by hydrolyzing asparagine, potently overcomes GBM cell resistance to TRAIL-induced extrinsic apoptosis [119]. Together, these preclinical observations suggest the therapeutic potential of combining TRAIL plus chemotherapeutic drugs in GBM treatment and encourage further preclinical and future clinical tests.

### 5.3. Combination with Non-Chemotherapeutic Drugs

Compared with chemotherapeutic drugs, efficacious synergistic effects of non-chemotherapeutic agents and TRAIL may be uncommon. However, evidence has indicated that lovastatin, a lipid-reducing drug, enhances TRAIL-induced GBM cell apoptosis synergistically [120]. Another example is salinomycin, an antibiotic used in the poultry industries to eliminate coccidiosis, which potentiates the cytotoxic effects of TRAIL on GBM cell lines [121]. Moreover, quinacrine is a small molecule antimalarial agent that was recently recognized with anticancer potentials [122], and it has been demonstrated that quinacrine is able to mediate the sensitization of GBM cells to TRAIL treatment [123], suggesting a combination treatment for GBM therapy. Other non-chemotherapeutic drugs exhibiting TRAIL-sensitizing activity include nelfinavir [124], troglitazone [125], digitoxin [126], melatonin [127], and Lanatoside C [128]. One of the limitations of these studies is a shortage of clarity regarding the molecular mechanisms accounting for the synergistic effects of these non-chemotherapeutic drugs and TRAIL, which require further investigations.

### 5.4. Combination with Other Inhibitors

Aside from the abovementioned inhibitors of the Akt-mTOR-S6K1 pathway, KPNB1, and PIM kinases, a large growing body of studies have also shown that a variety of inhibitors that do not belong to therapeutic drugs but sensitize GBM to TRAIL-induced apoptosis. For instance, histone deacetylase inhibitors (HDACIs), such as MS275, suberoylanilide hydroxamic acid and valproic acid, sensitize GBM cells to TRAIL-induced apoptosis in vitro and in vivo through c-myc-downregulated c-FLIP [129], suggesting the use of HDACIs in order to prime GBM for TRAIL-induced apoptosis by targeting c-FLIP. Since the anti-apoptotic Bcl-2 family members play a critical role in determining GBM sensitivity to TRAIL-induced apoptosis, inhibitors of this family members (BH3-mimetics), such as ABT-737 [130] and ABT-199 [131], were found to cooperate with TRAIL to induce apoptosis in several GBM cell lines in a highly synergistic manner. These results outline the antagonism of surviving machinery as a highly potent intervention to sensitize GBM cells to TRAIL combination treatment. Protein synthesis inhibitors, such as cycloheximide, can reverse the resistance of some cancer cells to TRAIL [132]. Two studies have revealed that the proteasome inhibitor bortezomib primes GBM, including GBM stem cells, for TRAIL sensitization, which is dependent on increased tBid stability, mitochondrial apoptosis, and modulation of the NF-κB signaling pathway [38,133]. Consistent with these reports, pretreating GBM with bortezomib potentiates natural killer cell cytotoxicity to induce TRAIL-mediated apoptosis and prolongs animal survival [134]. Taken together, these findings provide compelling evidence that the combination of bortezomib and TRAIL presents a promising strategy to promote TRAIL sensitization and trigger apoptosis in GBM.

## 6. Conclusions

Owing to excusive the tumoricidal property revealed by a large amount of pre-clinical and clinical studies, it is believed that targeting the TRAIL/TRAIL-R1/R2 axis holds great promise to be harnessed in combinatorial therapies for treating cancers, including GBM, a deadly cancer without efficacious therapeutic options. However, employing either recombinant human TRAIL or agonist antibodies against TRAIL-R1/2 to reactive the extrinsic apoptosis pathway in cancer cells for cancer therapy has yielded undesirable outcomes in previous clinical trials, casting a shadow over the future clinical applications of this strategy. Currently, seeking methods to overcome TRAIL resistance for enhancing TRAIL efficacy is a research focus. As with many cancers, the majority of GBM tumors are generally resistant to TRAIL-induced apoptosis largely due to several aberrations in genetics that result in low or loss of expression of apoptotic genes and simultaneous overexpression of anti-apoptotic genes, which comprise the TRAIL-induced apoptotic signaling pathway. Attempts to understand the mechanisms of TRAIL-induced apoptotic signaling in GBM have unraveled novel regulators in promoting or inhibiting TRAIL resistance, mainly through modulating the levels or activation of TRAIL-R1/R2, c-FLIP, caspase-8, and DISC. These studies provide novel therapeutic targets that can potentially interfere the resistance mechanisms to overcome GBM resistance to TRAIL-based therapies. Another strategy to improve the therapeutic efficacy of TRAIL and sensitize TRAIL resistance in GBM is through developing more effective approaches of delivering a sufficient amount of TRAIL to tumor lesions in the brain. Recent studies have shown the advantages of nanoparticles in increasing the delivery efficiency of TRAIL to sensitize GBM to TRAIL treatment. In addition, sensitizing GBM to TRAIL-induced apoptosis has proven effective by multiple preclinical studies through the combinatorial treatment of TRAIL with other agents, such as some commonly used chemotherapeutic and non-chemotherapeutic drugs and synthetic inhibitors. According to these progresses in overcoming TRAIL resistance in GBM, we expect more clinical trials will participate to test the therapeutic potency and safety of TRAIL-based combination modalities in GBM treatment. Finally, however, despite the abovementioned advances, how GBM tumors acquire TRAIL resistance is still not fully understood, and the mechanisms underlying synergistic effect of TRAIL and chemotherapeutic or non-chemotherapeutic drugs remain largely unexploited. Addressing these challenges is needed to overcome TRAIL resistance for maximizing the therapeutic potential of TRAIL in treating GBM.

## Figures and Tables

**Table 1:** Aberrations in TNF-related apoptosis-inducing ligand (TRAIL)-induced apoptotic signaling in GBM.

Genes	Encoded Proteins	Aberrations	Confirmed Resources	Effects	Ref
CASP8	Caspase-8	Gene promoter methylation	Clinic samples; cell lines	Inhibition of TRAIL-induced apoptosis	[56,63,64,65]
APAF1	Apaf-1	Loss of heterozygosity	Clinic samples	Inhibition of apoptosome assembly	[61]
TNFRSF10A	TRAIL-R1	Gene promoter methylation	Clinic samples; cell lines	Inhibition of TRAIL-induced apoptosis	[56,63]
TNFRSF10B	TRAIL-R2	Loss or structural aberration of gene	Cell lines	Inhibition of TRAIL-induced apoptosis	[62]
DIABLO	Smac	Loss or structural aberration of gene	Cell lines	Inhibition of TRAIL-induced apoptosis	[11,62]
BID	Bid	Loss or structural aberration of gene	Cell lines	Inhibition of TRAIL-induced apoptosis	[62]
PEA15	PEA-15	Possible altered protein stability	Clinic samples; cell lines	Blockage of death receptor activation	[56,66,67]
CFLAR	c-FLIP	Translational dysregulation	Cell lines	Inhibition of caspase-8	[57]
BCL2	Bcl-2	Transcriptional dysregulation	Clinic samples; cell lines	Inhibition of TRAIL-induced apoptosis	[58,59,68]
BCL2L1	Bcl-xL	Transcriptional dysregulation	Cell lines	Inhibition of TRAIL-induced apoptosis	[69]
MCL1	Mcl-1	Transcriptional dysregulation	Cell lines	Inhibition of TRAIL-induced apoptosis	[70,71]
XIAP	XIAP	Transcriptional dysregulation	Clinic samples	Inhibition of TRAIL-induced apoptosis	[72,73]
